# The Mobility Enhancement of Indium Gallium Zinc Oxide Transistors via Low-temperature Crystallization using a Tantalum Catalytic Layer

**DOI:** 10.1038/s41598-017-11461-0

**Published:** 2017-09-07

**Authors:** Yeonwoo Shin, Sang Tae Kim, Kuntae Kim, Mi Young Kim, Saeroonter Oh, Jae Kyeong Jeong

**Affiliations:** 1 Department of Electronic Engineering, Hanyang University, Seoul, 133-791 Republic of Korea; 2 Department of Materials Science and Engineering, Seoul National University, Seoul, 151-742 Republic of Korea; 3 Division of Electrical Engineering, Hanyang University, Ansan, Gyeonggi-do 15588 Korea

## Abstract

High-mobility indium gallium zinc oxide (IGZO) thin-film transistors (TFTs) are achieved through low-temperature crystallization enabled via a reaction with a transition metal catalytic layer. For conventional amorphous IGZO TFTs, the active layer crystallizes at thermal annealing temperatures of 600 °C or higher, which is not suitable for displays using a glass substrate. The crystallization temperature is reduced when in contact with a Ta layer, where partial crystallization at the IGZO back-channel occurs with annealing at 300 °C, while complete crystallization of the active layer occurs at 400 °C. The field-effect mobility is significantly boosted to 54.0 cm^2^/V·s for the IGZO device with a metal-induced polycrystalline channel formed at 300 °C compared to 18.1 cm^2^/V·s for an amorphous IGZO TFT without a catalytic layer. This work proposes a facile and effective route to enhance device performance by crystallizing the IGZO layer with standard annealing temperatures, without the introduction of expensive laser irradiation processes.

## Introduction

Amorphous metal oxide semiconductor thin-film transistors (TFTs) are used as the backplane electronics in liquid crystal displays (LCDs) and organic light-emitting diode (OLED) displays, for their field-effect mobility >10 cm^2^/V·s, good uniformity over large glass substrates sizes, and low temperature process^1–4^. Conventionally, amorphous silicon (*a*-Si:H) has been the well-established standard backplane technology due to its low cost, good size scalability, and excellent manufacturability^5^. However, *a*-Si:H suffers from a low charge carrier mobility of less than 1 cm^2^/V·s. Low-temperature poly-silicon (LTPS) devices with carrier mobility values greater than 70 cm^2^/V·s are used in mobile device applications. LTPS, which is primarily formed via the crystallization of *a*-Si:H using excimer laser annealing (ELA), enables high-resolution and narrower display borders with integrated gate driver circuits^6–8^. However, the high equipment and manufacturing costs, as well as the difficulty of scaling ELA equipment beyond generation 6 glass substrate sizes have limited its application in large screen displays^8, 9^.

As the demand continuously grows for larger screens, higher resolution, higher frame rates, and stereoscopic vision for future glasses-free 3-dimensional displays or virtual reality head-mount displays, requirements for high TFT mobility and low RC delays have become crucial^10^. High carrier mobility allows faster charge and discharge of the pixel storage capacitor, which results in less signal processing time for each selected line of the pixel array. Furthermore, the reduction of TFT dimensions for the same drive current leads to larger aperture ratios and lower power consumption^11^.

Much research effort has been made toward identifying a high-device performance metal-oxide semiconductor candidate. Generally, high In-content oxide materials have higher electron carrier concentration and superior field-effect mobility, but suffer from bias instability under illumination conditions^12, 13^. Other Sn-based materials^14, 15^, ZnON^16, 17^, and bi-layer active layer structures^18–21^ have been studied. Despite these research efforts, InGaZnO (In:Ga:Zn = 1:1:1 atomic ratio) is the most commonly used oxide material in manufacturing due to its good uniformity, low process temperature, and relatively good bias-thermal stress (BTS) stability and chemical stability. Hence, the implementation of innovative TFT structures and processes into current technology to boost the device performance of the industry-standard InGaZnO would be economically beneficial.

Nomura *et al*. reported a single-crystal IGZO transistor with a field-effect mobility of 80 cm^2^/V·s using thermal annealing at 1400 °C for 30 minutes^22^. IGZO deposited at room temperature begins to crystallize in a polycrystalline state at temperatures of 600~700 °C via thermal annealing^23–26^ or local heating using excimer laser irradiation^27, 28^. Although the onset temperature of crystallization may differ depending on the deposition method, argon/O_2_ ratio, active layer thickness, and annealing ambient^29^, the necessary temperatures are far too high to be used for devices fabricated on glass substrates. Moreover, the grain boundaries in polycrystalline IGZO may form energy barriers that impede the charge carrier conduction, sometimes resulting in degradation of electron mobility to values lower than that of *a*-IGZO. Yamazaki *et al*. deposited an IGZO layer by sputtering onto a heated substrate of *ca*. 300 °C to form c-axis alignment crystalline IGZO^30, 31^, followed by a 450 °C annealing step to ensure uniform film quality and an increased portion of c-axis-aligned crystalline regions. In this case, the difference in angle between adjacent grains was small and changed gradually across grain boundaries^30, 32^. Nevertheless, the field-effect mobility value was below 10 cm^2^/V·s. In this regard, a low-temperature crystallization technique that substantially enhances the mobility of IGZO TFTs would be useful in next-generation display applications.

In this work, we apply a tantalum (Ta) metal capping layer to the IGZO back surface and subject it to various annealing temperatures. Previously the thermal annealing of Ta/zinc tin oxide (ZTO) stack was found to cause the low temperature crystallization, which allowed the promising enhancement of the field-effect mobility in the resulting ZTO TFT^33^. However, it is noted that the industry standard IGZO materials is anticipated to be hardly replaced by the ZTO semiconductor because of the difficulty in the fabrication of its large-size target (≥8 Gen.) and wet etching as well as the optimization of the overall process. Thus, the industry standard IGZO channel was chosen for the facile implantation in this study. During annealing, the Ta layer becomes oxidized while facilitating partial crystallization of the IGZO layer at temperatures as low as 300 °C. Metal-induced crystallization of the IGZO active layer at 300 °C results in a TFT with respectable device characteristics including a field-effect mobility of 54.0 cm^2^/V·s, subthreshold slope of 0.3 V/decade, and threshold voltage of 0.2 V. The proposed device structure and process can be straightforwardly implemented into existing IGZO backplane technology to boost device performance using a crystallization technique that requires neither high-temperature annealing nor excimer laser irradiation.

## Results

**Figure 1:**
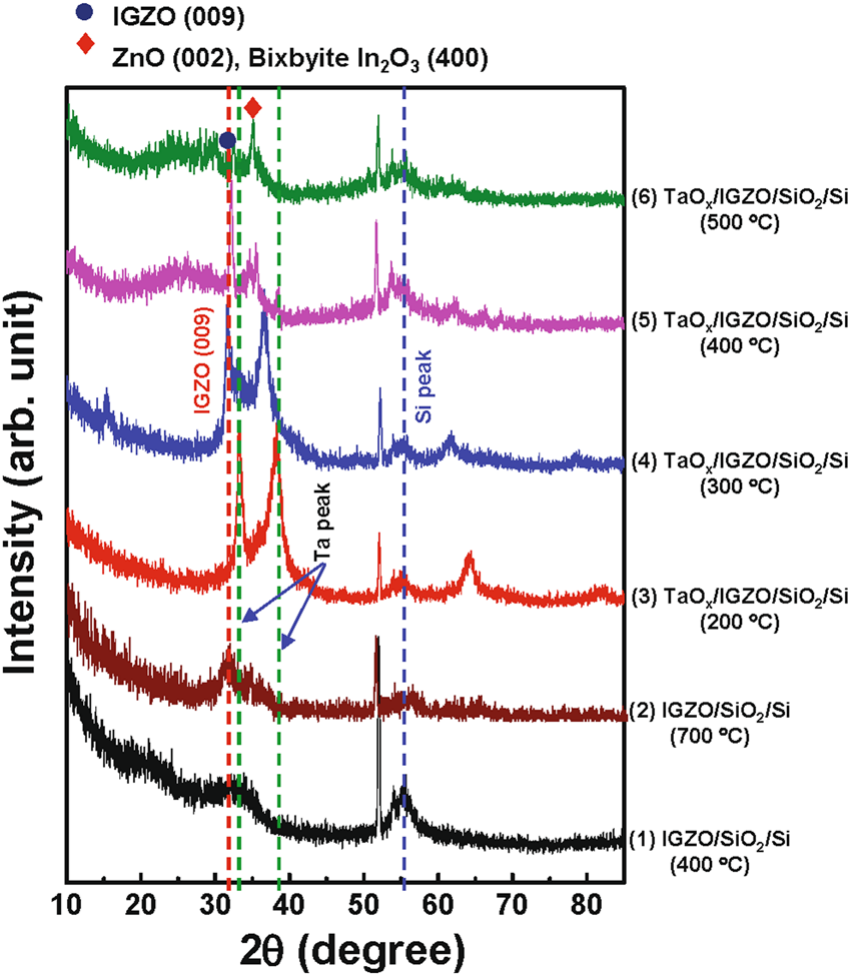
XRD spectra of the IGZO /SiO_2_/Si stack with and without the Ta catalytic layer, after annealing at various temperatures under O_2_ atmosphere.

The effects of the Ta catalytic layer and annealing temperature on the crystallographic nature of IGZO films are examined in detail. Figure 1 shows the X-ray diffraction (XRD) spectra of the IGZO/SiO_2_/Si stack both with and without the Ta layer at various annealing temperatures under O_2_ atmosphere. The XRD peak near 56°, common across all samples, comes from the Si substrate (Fig. S2 in Supplementary Information (*SI)*). After annealing of the a-IGZO/SiO_2_/Si film at 400 °C, only a broad pattern near ~33° can be observed with no sharp diffraction peaks, indicating that the IGZO film remains amorphous. As expected, at a higher annealing temperature of 700 °C, the XRD spectrum shows a discernible (009) diffraction peak. This indicates that the IGZO layer has crystallized, which agrees with previous reports in the literature^34^. Next, the Ta layer is applied on top of the amorphous IGZO layer and annealed at temperatures ranging from 200 to 500 °C. At 200 °C, a set of strong diffraction peaks at 2θ = 33.7 and 38.5° corresponds to the tetragonal β-Ta film (002) and (110) peaks, respectively (also see Fig. S2 in *SI*). When the annealing temperature is further increased to 300 °C, the Ta XRD peaks are shifted toward a lower angle, as shown in Fig. S2. This may be caused by an increase in lattice spacing due to the increased thermal stress in the metallic film. A distinct peak near 33° can be observed and is attributed to the IGZO (009) peak seen from the (4) spectrum, though it is not as prominent. Lattice ordering and thus partial crystallization occur near the Ta/IGZO interface, which will be discussed later. Note that the onset temperature of IGZO crystallization is reduced by more than 300 °C through the incorporation of the Ta catalytic layer. At an annealing temperature of 400 °C, a sharp IGZO (009) peak and IGZO (104), (015) peaks can be seen, indicating a well-defined crystalline state in the IGZO layer. Simultaneously, the metallic Ta peaks are no longer present, suggesting the formation of tantalum oxide (TaO_*x*_) via oxidation of the Ta layer in the ambient O_2_ (also see Fig. S2). At annealing temperatures of 500 °C, the crystalline IGZO peaks decrease, while a sharp peak at 2θ = 35° appears, which is assignable to either ZnO (002) or the bixbyite In_2_O_3_ (400) phase. This result suggests that, at high annealing temperatures, the weakened cation bonds prefer to form small grains of ZnO or In_2_O_3_ rather than rearrange into crystalline grains of InGaZnO_4_. The microscopic structure of the Ta/IGZO/SiO_2_/Si film at annealing temperatures of 200, 300, and 400 °C was further examined.

**Figure 2:**
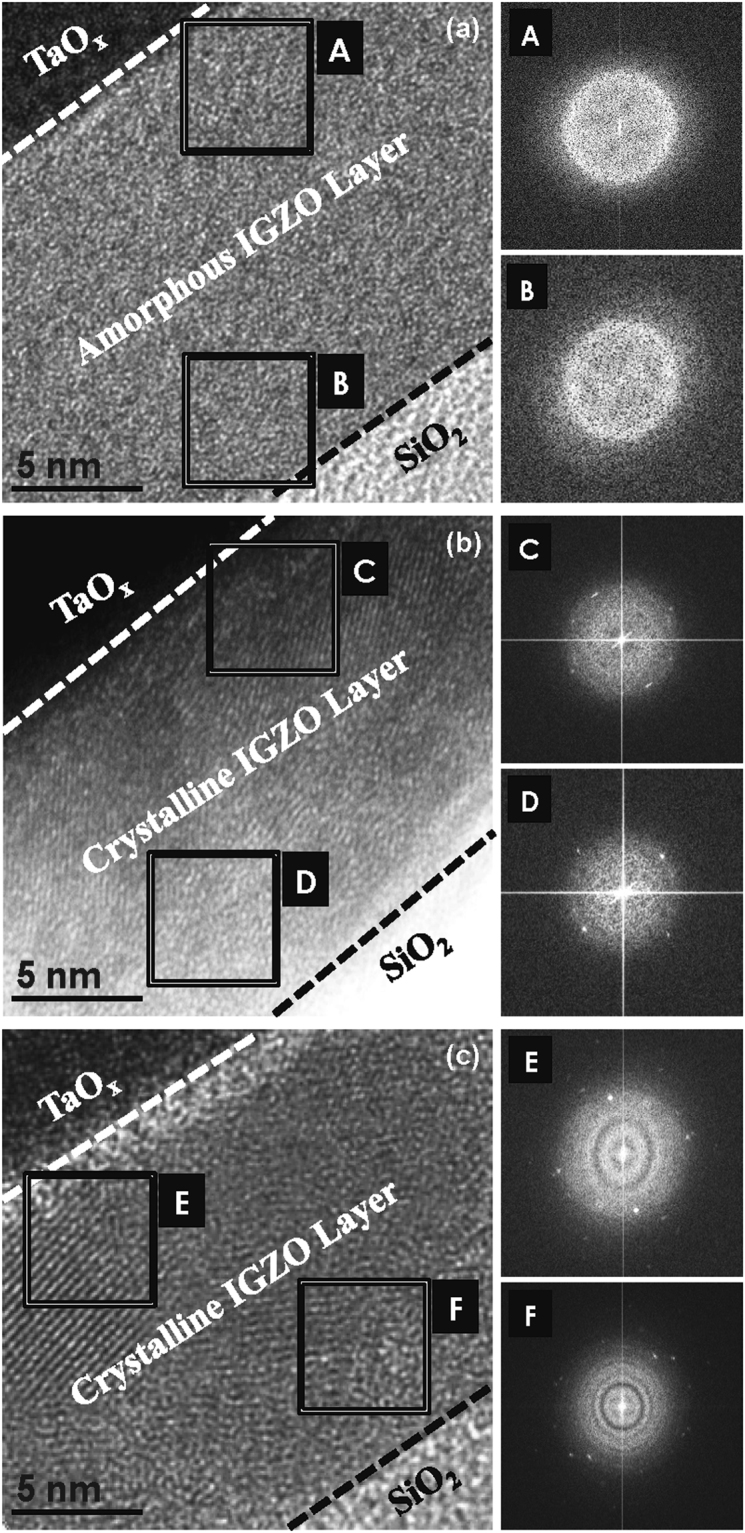
Cross-sectional TEM images of the IGZO layer with the Ta catalytic layer after thermal annealing at (**A**) 200 °C, (**B**) 300 °C, and (**C**) 400 °C under O_2_ atmosphere. Selected area electron diffraction (SAED) patterns near the top and bottom interface are shown in the insets.

Figure 2 shows cross-sectional transmission electron microscopy (TEM) images of the IGZO layer with the Ta catalytic layer after thermal annealing under O_2_ atmosphere. No signs of crystalline regions appear near the Ta/IGZO interface (denoted as A) after annealing at 200 °C, nor near the channel region (denoted as B), as shown in Fig. 2(A). Selected area electron diffraction (SAED) patterns also exhibit diffuse rings that indicate an amorphous phase. Figure 2(B) shows a TEM image of the Ta/IGZO/SiO_2_ film after annealing at 300 °C. Slight indications of ring patterns and bright spots appear near both the top and bottom interfaces of the IGZO layer (denoted as C and D, respectively), indicating the occurrence of small-grain crystallization throughout the entire active layer. The Ta atoms induce a change in the bonding characteristics of the IGZO, resulting in the rearrangement of the constituent atoms to form crystalline regions. The TEM images of the samples annealed at 400 °C clearly show diffraction rings with spot patterns in the areas denoted as E and F in Fig. 2(C), indicating that the entire IGZO active layer is crystallized with larger grains than the sample annealed at 300 °C. The SAED patterns show that the crystal structure is more organized at the IGZO/SiO_2_ interface. Once the Ta-induced crystalline regions near the back-interface were established, the crystalline regions grew and propagated in the depth direction, free of Ta atoms (see Fig. S6 in *SI*).

**Figure 3:**
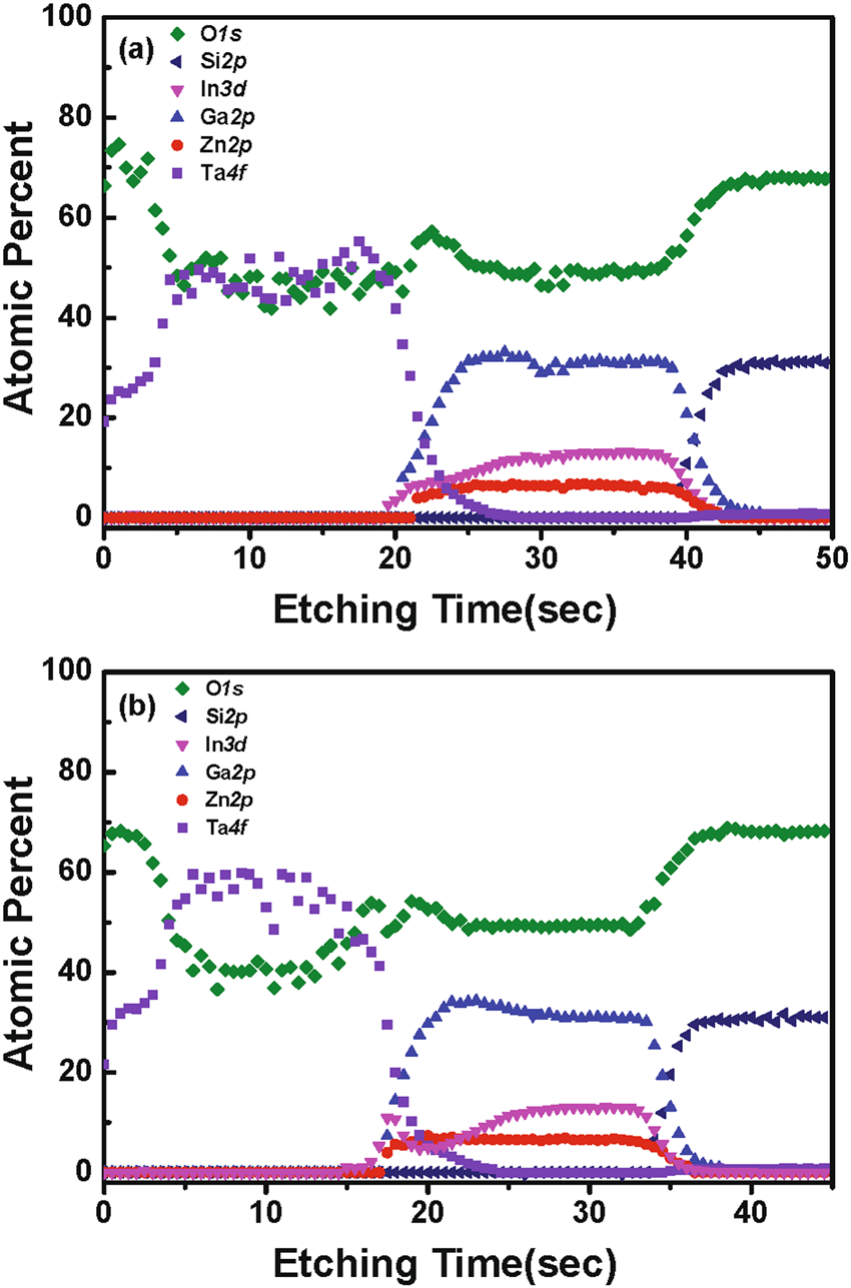
Depth profile of samples annealed at (**a**) 200 °C and (**b**) 300 °C, obtained from XPS analysis.

**Figure 4:**
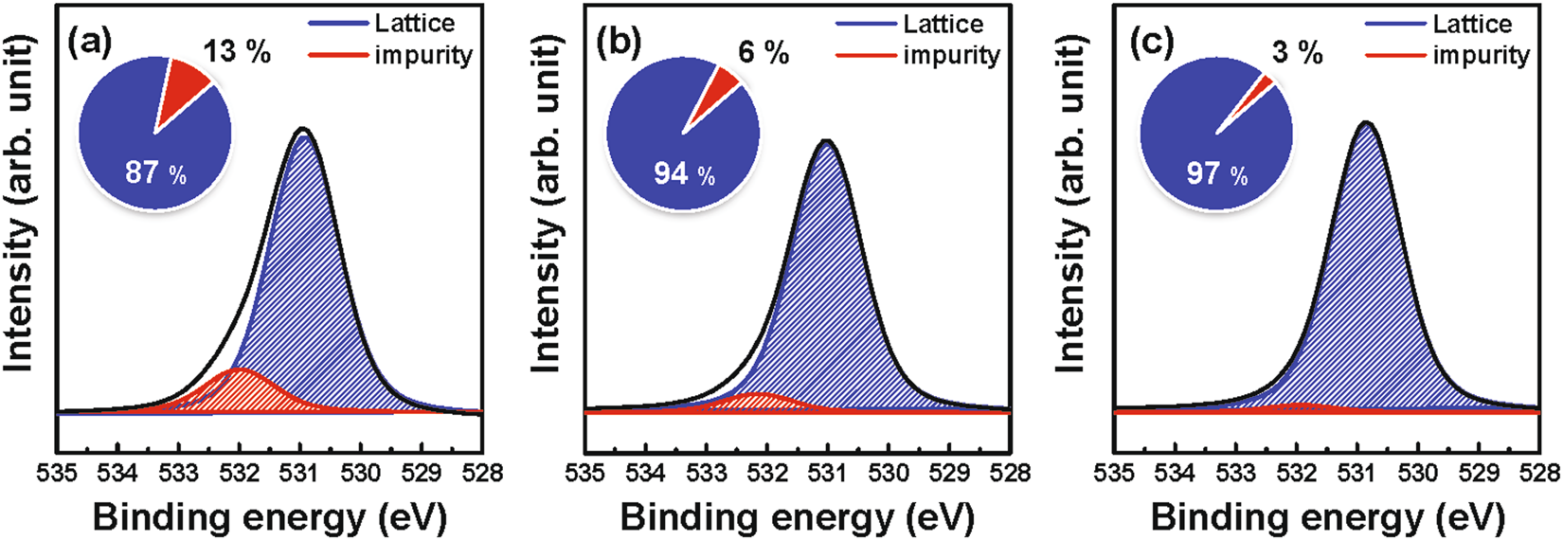
O *1 s* XPS spectra of the (**a**) reference IGZO sample, (**b**) Ta/IGZO sample annealed at 200 °C, and (**c**) Ta/IGZO sample annealed at 300 °C.

X-ray photoelectron spectroscopy (XPS) analysis was performed to reveal the distribution of elements within the material stack along the depth direction, as well as the chemical binding states of the constituent elements. Figure 3 shows the XPS depth profile of the samples annealed at 200 and 300 °C under O_2_ atmosphere. The Ta layer is oxidized to form TaO_*x*_ after thermal annealing. As shown in Fig. 3(b), there is a slight increase of In and Ga elements at the interface in the Ta layer after annealing at 300 °C. Figure 4 shows the O 1 *s* XPS spectra of the reference IGZO film without the catalytic layer and Ta/IGZO stacks annealed at 200 and 300 °C, respectively, which were obtained from depth profiling XPS analysis. The subpeaks at 530.9 and 532.0 eV are assigned to the oxygen bonded to fully-coordinated metal ions (M-O lattice) and hydroxyl group-related oxygen bonds, respectively^35, 36^. The M-O lattice portion of the Ta/IGZO film annealed at 300 °C increased from 87% of the value of the reference IGZO film to 97%. Conversely, the hydroxyl group-related portions decreased from 13% of the value of the reference IGZO film to 3%.

It is interesting to discuss how the Ta catalytic layer and subsequent annealing affect the structural and electrical properties of the semiconducting IGZO film. The thermal oxidation of Ta on the IGZO channel layer during the post deposition annealing (PDA) process clearly affects the chemical states of the underlying IGZO film. The Gibbs free energies of formation (*ΔG*_*f*_) for In_2_O_3_, Ga_2_O_3_, ZnO, and Ta_2_O_5_ are −830.7, −998.3, −348.1, and −1911.2 kJ/mol, respectively^37, 38^. The lower *ΔG*_*f*_ of Ta_2_O_5_ indicates that Ta atoms have stronger oxidation tendencies than those of In_2_O_3_, Ga_2_O_3_, and ZnO (and hence, IGZO). Therefore, it is reasonable that the PDA of the Ta/IGZO stack at an elevated temperature (>500 °C) will cause oxidation of the Ta film and the simultaneous reduction of the IGZO film near the Ta/IGZO interface regions, which involves the elimination of the lattice oxygens bonded to In, Ga, and Zn cations. However, the lower PDA temperature (~300 °C) in this study will kinetically hinder the reduction reaction because breaking cation-to-oxygen bonds (In-to-O, Ga-to-O, or Zn-to-O) requires a high activation energy (>1 eV)^39^. In this case, the weakly bonded oxygen species, such as interstitial oxygen and the hydroxyl groups in the IGZO film, will preferentially be eliminated and consumed during the formation of TaO_x_^40^. Indeed, some loosely-bonded oxygen species were calculated to exist in the form of OH impurities in the metal oxide semiconductor^41^. This interpretation is consistent with the reduction of the impurity-related oxygen peak in the O 1 *s* XPS spectrum of the Ta/IGZO films.

**Figure 5:**
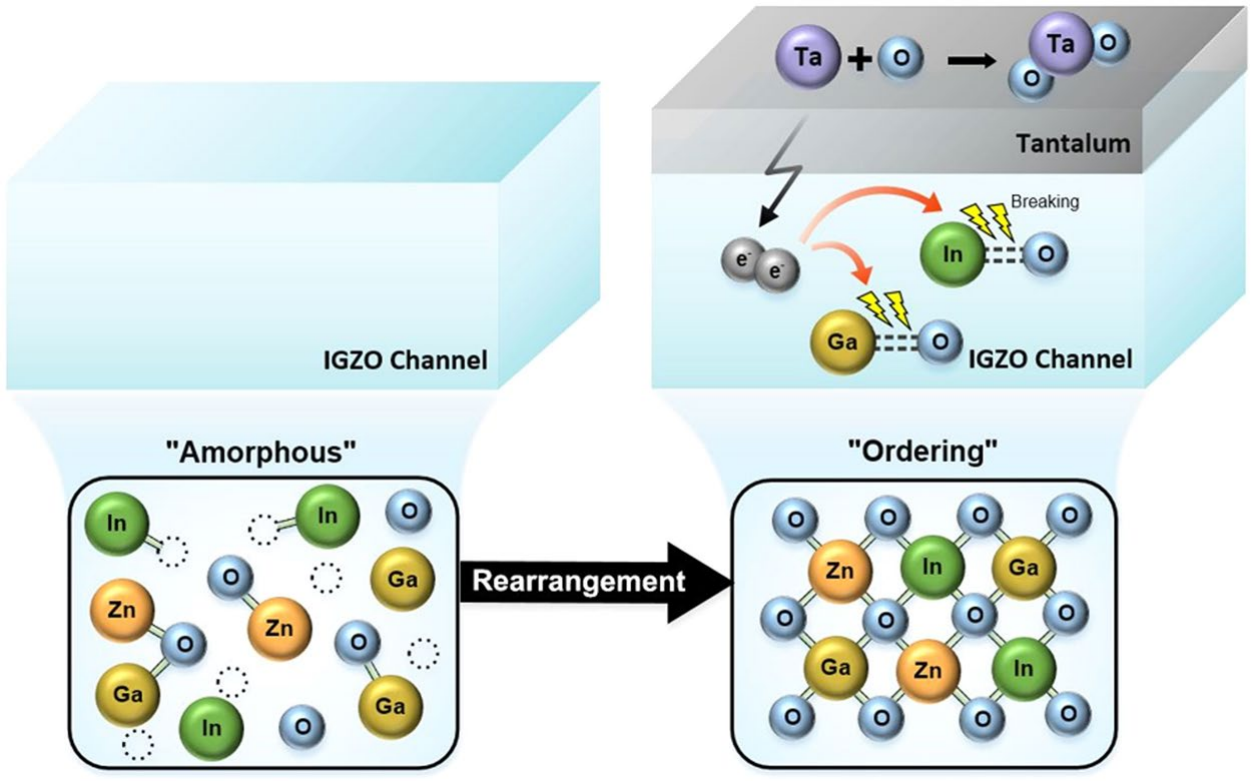
Schematic representation of the IGZO crystallization mechanism induced by the Ta catalytic layer.

Figure 5 depicts a schematic representation of the Ta-induced crystallization of IGZO near the top interface. Unlike the numerous metal-induced crystallization studies on a-Si:H^42^, there are few reports on the metal-induced crystallization of metal-oxides. Yang *et al*. reported that the crystallization temperature of TiO_2_ films can be reduced by using a Ni contact layer^43^. Temperatures in the range of 200–300 °C are not sufficient to promote the diffusion of metal interstitials in a metal-oxide material. Rather, the Ni atoms assist the crystallization of TiO_2_ through an intermediate reaction. Following a similar model, the Ta layer can release electrons into the underlying IGZO layer. These electrons are transferred to the anti-bonding orbitals of M-O (M = In, Ga) bonds that weaken the M-O bonds. During the subsequent thermal annealing step, the weakened M-O bonds are likely to be broken. Indeed, the metallic In and Ga elements between TaO_x_ and IGZO films are still observed after the PDA of 200 and 300 °C, as shown in the In *3d* XP (Fig. S3 in *SI*) and Ga *2p* XP spectra (Fig. S4 in *SI*), respectively. The rearrangement and local diffusion of broken M-O bonds in conjunction with the atomic In and Ga may be the reason for the low-temperature crystallization of IGZO film.

**Figure 6:**
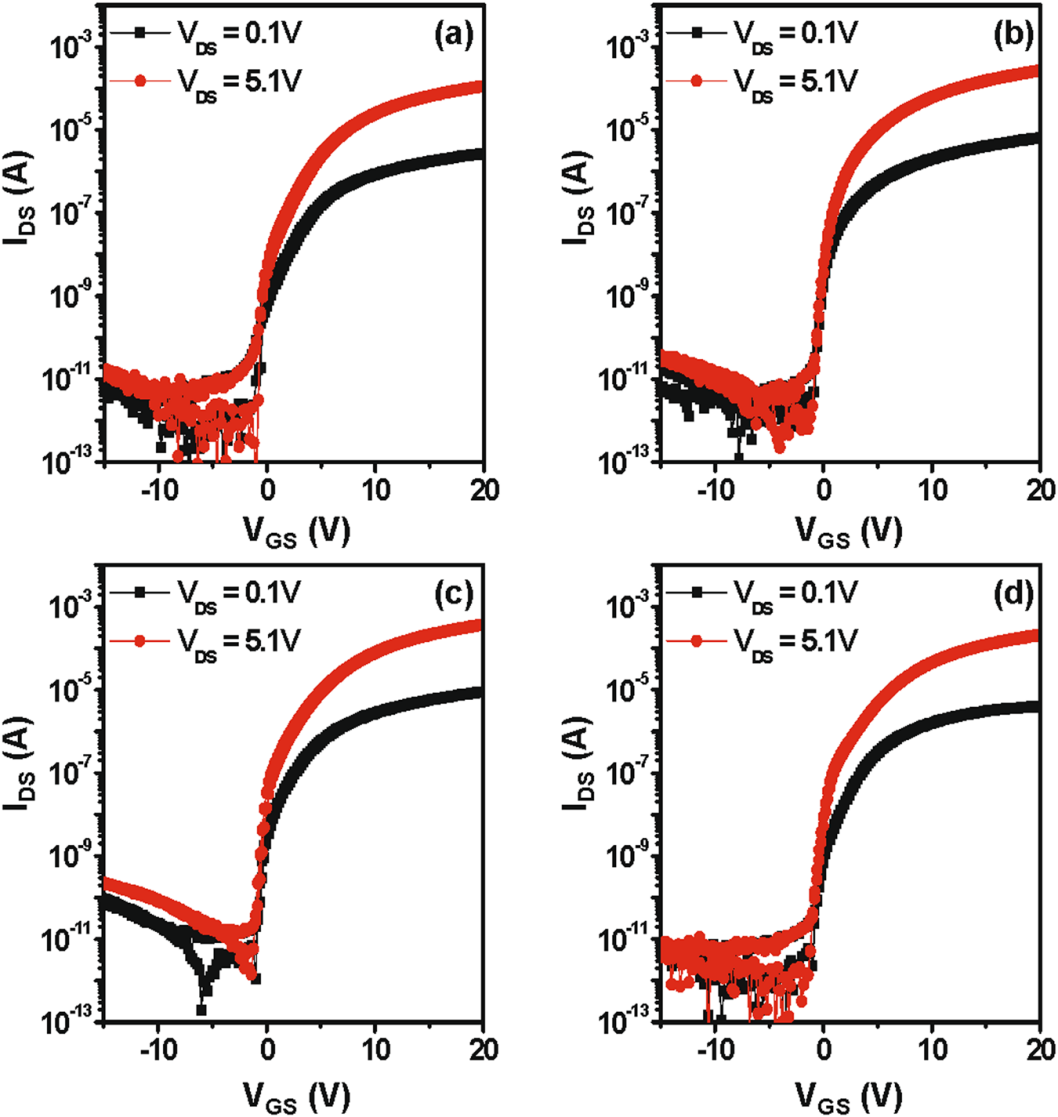
Transfer characteristics (*I*_D_ − *V*_GS_) of the (**a**) reference IGZO device without the catalytic layer and the Ta-induced polycrystalline IGZO TFT annealed at (**b**) 200 °C, (**c**) 300 °C, and (**d**) 400 °C under O_2_ atmosphere. The thickness of the IGZO films for all IGZO devices was ~15 nm.

**Figure 7:**
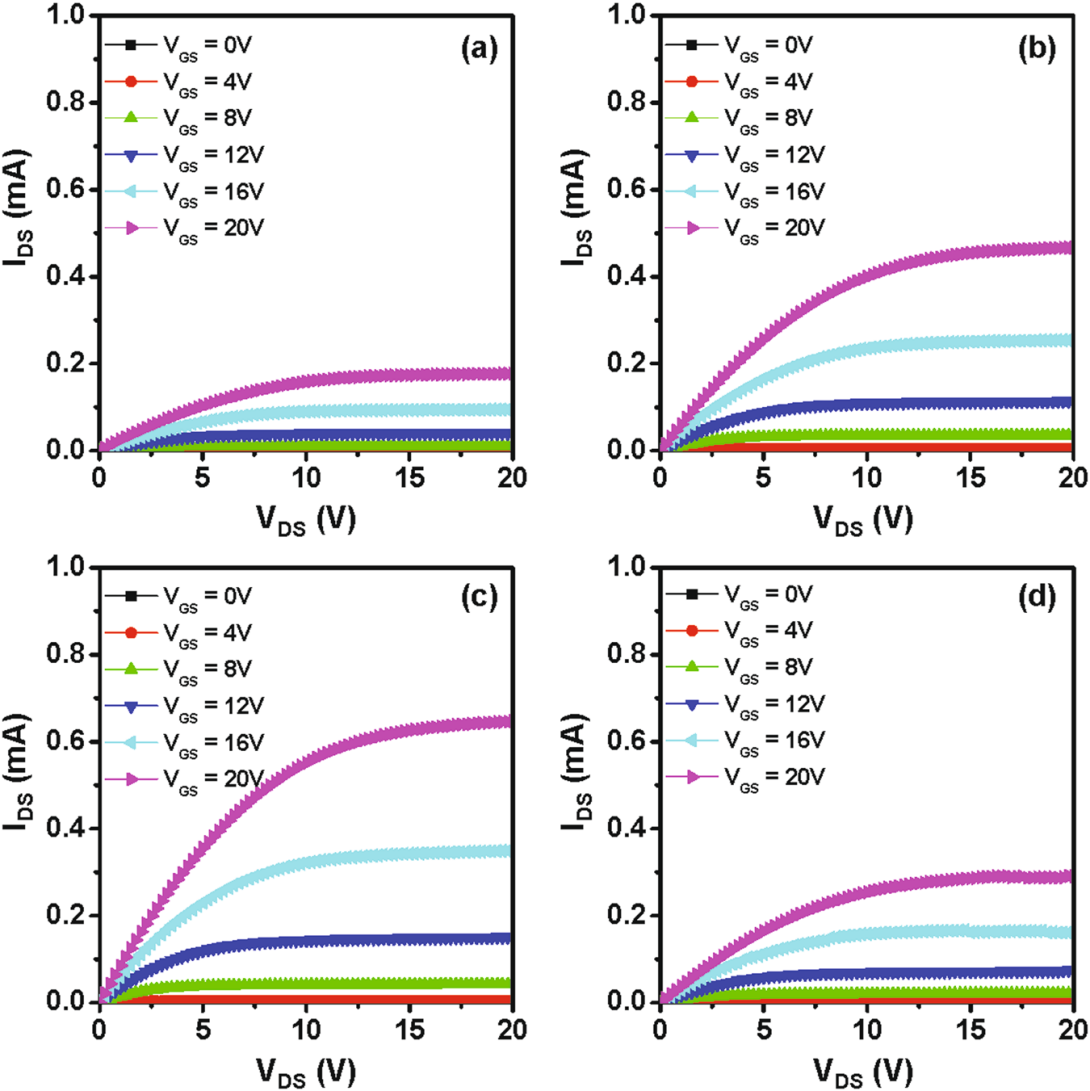
Output curves (*I*_D_ − *V*_DS_) of the (**a**) reference IGZO device without the catalytic layer and the Ta-induced polycrystalline IGZO TFT annealed at (**b**) 200 °C, (**c**) 300 °C, and (**d**) 400 °C under O_2_ atmosphere.

Figure 6 shows the representative transfer characteristics (*I*_D_−*V*_GS_) of the reference IGZO device without the catalytic layer and the Ta-induced polycrystalline IGZO TFT. The threshold voltage (***V***_***TH***_) is taken as the voltage where *I*_D_ = W/L × 10 nA at *V*_DS_ = 5.1 V. The field-effect mobility (***μ***_***FE***_) is calculated via the maximum peak value at a *V*_DS_ of 0.1 V. The subthreshold gate swing (***SS***) is taken from the transfer curve at *V*_DS_ = 0.1 V. The device characteristics are listed in Table 1. The reference IGZO device has ***μ***_***FE***_ = 18.1 cm^2^/V·s, ***V***_***TH***_ = 0.9 V, and ***SS*** = 0.8 V/decade. The Ta-induced crystallization process causes the resulting IGZO TFTs to have a higher ***μ***_***FE***_ value and lower ***V***_***TH***_ value. At a PDA temperature of 300 °C, the devices exhibited a remarkable ***μ***_***FE***_ of 54.0 cm^2^/V·s, which is a 3-fold improvement relative to the reference device; however, the device subjected to annealing at 400 °C exhibits mobility degradation at ***μ***_**FE**_ = 27.3 cm^2^/V·s. Although the ordering of IGZO atoms helped to improve the electron carrier transport, an excess of grain boundaries may have adverse effects by presenting numerous energy barriers in the conduction path. Therefore, optimum device characteristics are achieved when some crystallization occurs, but not enough to generate grain boundaries that are detrimental to the current flow. The optimum annealing temperature in this study is 300 °C. Figure 7 shows the output characteristics (*I*_D_ − *V*_DS_) of the four device cases, which exhibit good drain conductance that agrees with the transfer curves. For a bottom-gate, top-contact structure, the entire back-channel cannot be deposited with a metallic layer as it will short the source and drain electrodes. Thus, the current will flow through a-IGZO regions on either sides of the polycrystalline region where the Ta layer is applied. It is suggested that the metal layer can extend along the entire channel length in a bottom-gate, bottom-contact device structure to fully take advantage of the Ta-induced crystallization effect. It would be interesting to note the role of O_2_ atmosphere on the Ta-induced crystallization of a-IGZO films. The samples annealed at 300 and 400 °C under N_2_ atmosphere also showed the similar crystallization as shown in Fig. S7 in *SI*. Furthermore, the Ta-induced polycrystalline IGZO TFTs annealed at 300 and 400 °C under N_2_ atmosphere exhibited the improved ***μ***_***FE***_ value mobilities (see Fig. S8 and Table S1 in *SI*), indicating that the existence of O_2_ atmosphere is not a critical factor on the Ta-induced low-temperature crystallization of a-IGZO films.

**Table 1:** Summary of the TFT device parameters with the reference IGZO and Ta/IGZO annealed at various temperatures.

Samples	μ_FE_ (cm^2^/Vs)	SS (V/decade)	V_TH_ (V)	I_ON/OFF_
(a) Control Device	18.1 ± 0.6	0.8 ± 0.1	0.9 ± 0.2	1.2 × 10^7^
(b) Ta/ IGZO 200 °C	42.7 ± 2.7	0.4 ± 0.1	0.5 ± 0.2	3.4 × 10^7^
(c) Ta/ IGZO 300 °C	54.0 ± 4.7	0.3 ± 0.1	0.2 ± 0.2	4.4 × 10^7^
(d) Ta/ IGZO 400 °C	27.3 ± 1.1	0.5 ± 0.1	0.7 ± 0.3	2.2 × 10^7^

Average and standard deviation values are included.

**Figure 8:**
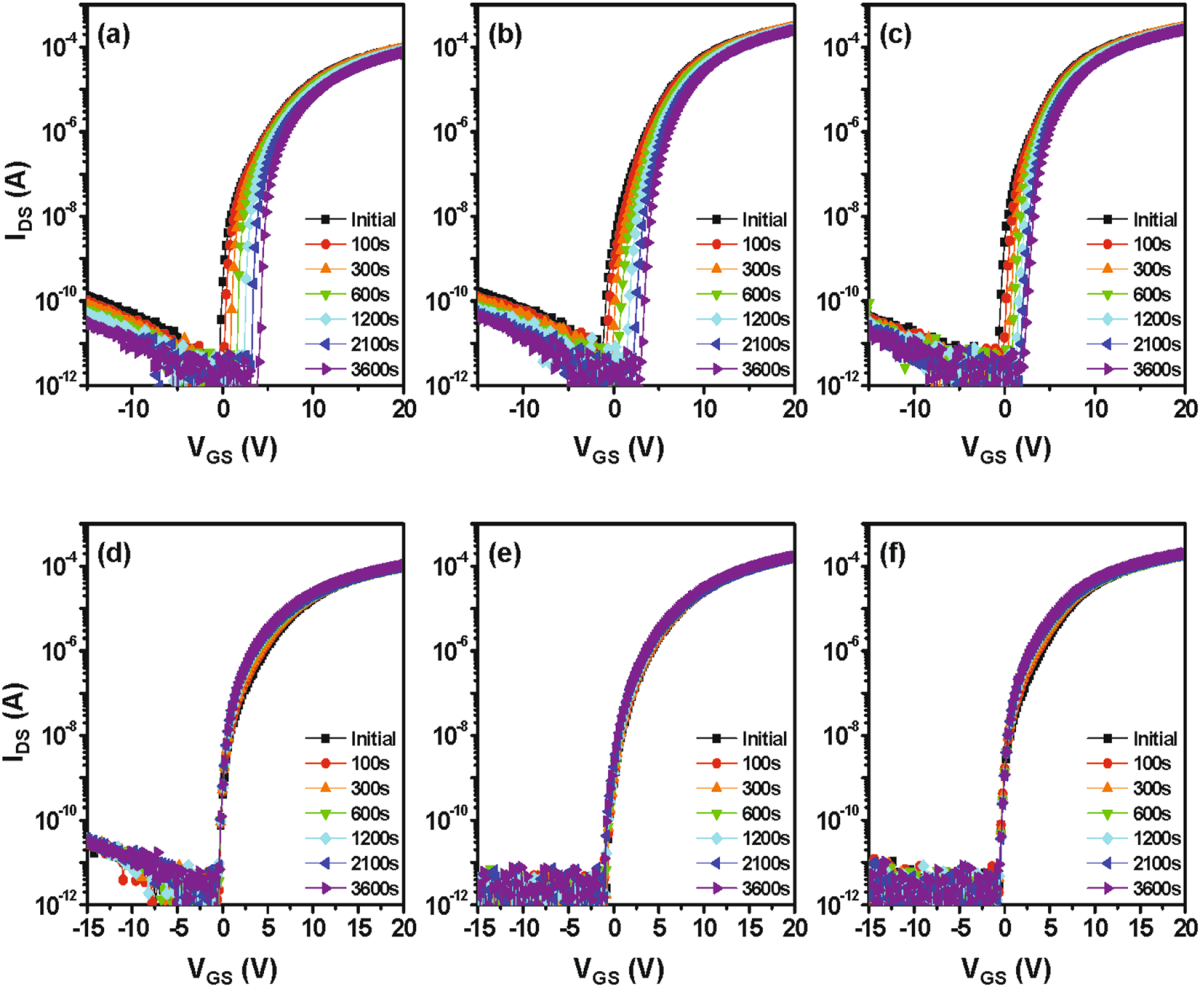
Evolution of transfer characteristics under PBS for the (**a**) reference IGZO device and the (**b**) Ta-induced polycrystalline IGZO TFT annealed at (**b**) 200 °C and (**c**) 300 °C under O_2_ atmosphere. NBS stability characteristics are shown in (**d**), (**e**), (**f**). The PBS (NBS) stress conditions are *V*_*GS*_ = *V*_*TH*_ + 20 V (*V*_*GS*_ = *V*_*TH*_ − 20 V) and *V*_*DS*_ = 5.1 V.

Figure 8 shows the stability characteristics of the IGZO TFTs under 1 hour of positive gate bias stress (PBS) or negative gate bias stress (NBS). The PBS conditions are *V*_GS_ = *V*_*TH*_ + 20 V and *V*_DS_ = 5.1 V, while the NBS conditions are *V*_GS_ = *V*_*TH*_ − 20 V and *V*_DS_ = 5.1 V. The threshold voltage shift (**Δ** ***V***_**TH**_) under PBS for the IGZO TFT with the Ta layer annealed at 300 °C improves to 2.65 V compared to 3.35 V of the IGZO reference TFT. The superior reliability of the crystallized IGZO TFTs at 300 °C is consistent with the reduced defects density as a result of high-degree lattice ordering and the impurity scavenging effect. Under NBS, all devices show comparable stability characteristics with Δ*V*_th_ of −0.5 V.

## Discussion

We investigated the effects of Ta-induced, low-temperature crystallization of IGZO films on TFT characteristics. The presence of a Ta layer on the IGZO active layer results in the onset annealing temperature for crystallization being lowered from 600 °C to 300 °C. Metal Ta film is oxidized to form TaO_*x*_ during the thermal annealing step. XRD and TEM analyses show partial crystallization at 300 °C and larger crystal grains throughout the total active layer at 400 °C. Ta acts as a catalyst to break weak IGZO bonds, where the broken bonds within IGZO are rearranged to form crystallized regions during thermal annealing. The intermediate crystallization region serves as a nucleation site for the crystalline regions to grow toward the bottom interface. Bottom-gate structure TFT devices are fabricated using the Ta layer formed on top of the active layer between the source and drain electrodes. Through the use of this material stack, the field-effect mobility is significantly increased from 18 cm^2^/V·s (of a device without the catalytic Ta layer) to 54.0 cm^2^/V·s at 300 °C annealing. However, further annealing at temperatures higher than 400 °C introduced grain boundaries throughout the active layer, which hampered the electron transport and hence decreased the mobility. Metal-induced crystallization and subsequent annealing at 300 °C provides an effective method to significantly enhance the device performance of standard IGZO TFTs on glass or plastic substrates with a process temperature constraint (<400 °C).

## Methods

Bottom-gate, top-contact structure IGZO devices are fabricated for this study. A 100-nm SiO_2_ layer is grown via thermal oxidation on a heavily-doped p-type Si wafer. The highly-doped Si substrate acts as the gate electrode, while the SiO_2_ layer serves as the gate insulator. A 15-nm a-IGZO (In:Ga:Zn = 1:1:1 at. %) active layer is deposited via RF sputtering under an Ar atmosphere and patterned using a shadow mask. The RF power of the IGZO target is 100 W, and the chamber pressure is fixed at 3 mTorr. Source/drain electrodes are formed via the DC sputtering of ITO. During deposition of the S/D electrode, the working pressure is 5 mTorr under an Ar atmosphere, and the DC power of the ITO target is 50 W. The channel width and length of the IGZO TFT are 1000 μm and 300 μm, respectively. A post-deposition annealing (PDA) step was performed at 400 °C for 1 hour under an O_2_ atmosphere. A 20-nm-thick Ta thin film, serving as the crystallization catalytic layer, is sputtered selectively through a shadow mask on top of the active layer between the source and drain electrodes with dimensions of W / L = 2300 μm / 150 μm (see Fig. S1 in ***SI***). A final annealing step with temperatures varying from 200 to 500 °C is performed in ambient O_2_ or N_2_. Device channel width (W) and length (L) of 1000 and 300 μm, respectively, are used throughout this study.

XRD analysis is carried out using a step scan mode with a step size of 0.02° (2θ), 0.3 s per step, and Cu-Kα radiation (40 kV, 30 mA). Cross-sectional transmission electron microscopy (TEM, Tecnai F20 ultra-high resolution TEM operating at 200 kV) analysis is performed on the prepared Ta/IGZO film samples to examine the local crystal structure within the active layer. Using X-ray photoelectron spectroscopy (XPS, SIGMA PROBE, ThermoG, UK), we investigate the chemical binding states of the Ta/IGZO films as well as the atomic composition profile along the depth direction by sputtering Ar^+^ ions of 1 keV energy. Electrical measurements of the TFT characteristics are performed using a Keithley 2636 source parameter analyzer at room temperature in ambient air.

## Electronic supplementary material

Supporting Information
