# Author Correction: The Mobility Enhancement of Indium Gallium Zinc Oxide Transistors via Low-temperature Crystallization using a Tantalum Catalytic Layer

**DOI:** 10.1038/s41598-018-25896-6

**Published:** 2018-05-15

**Authors:** Yeonwoo Shin, Sang Tae Kim, Kuntae Kim, Mi Young Kim, Saeroonter Oh, Jae Kyeong Jeong

**Affiliations:** 1 Department of Electronic Engineering, Hanyang University, Seoul, 133-791 Republic of Korea; 2 Department of Materials Science and Engineering, Seoul National University, Seoul, 151-742 Republic of Korea; 3 Division of Electrical Engineering, Hanyang University, Ansan, Gyeonggi-do 15588 Korea

Correction to: *Scientific Reports* 10.1038/s41598-017-11461-0, published online 07 September 2017

This Article contains an error in the Acknowledgements section.

“This study was supported by the Industrial Strategic Technology Development Program funded by MKE/KEIT under Grant 10048560 and by a National Research Foundation (NRF) grant funded by the Korean government (NRF-2015R1A2A2A01003848).”

should read:

“This study was supported by the Industrial Strategic Technology Development Program funded by MKE/KEIT under Grant 10051403 and by a National Research Foundation (NRF) grant funded by the Korean government (NRF-2015R1A2A2A01003848).”

