# Supplementary material for: The Mobility Enhancement of Indium Gallium Zinc Oxide Transistors via Low-temperature Crystallization using a Tantalum Catalytic Layer
Source: Sci Rep. 2017 Sep 7;7:10885. doi: 10.1038/s41598-017-11461-0 (PMC5589867; doi:10.1038/s41598-017-11461-0)
Supplement: Supplementary file 1 — Supporting Information [file 41598_2017_11461_MOESM1_ESM.pdf]

Supplementary Information

The Mobility Enhancement of Indium Gallium Zinc Oxide Transistors via Low-temperature Crystallization using a Tantalum Catalytic Layer

Yeonwoo Shin[1], Sang Tae Kim[1], Kuntae Kim[2], Mi Young Kim[2], Saeroonter Oh[3], and Jae Kyeong Jeong[1]**

[1] Department of Electronic Engineering, Hanyang University, Seoul 133-791, Republic of Korea

[2] Department of Materials Science and Engineering, Seoul National Univerity, Seoul 151-742, Republic of Korea

[3] Division of Electrical Engineering, Hanyang University, Ansan, Gyeonggi-do, 15588, Korea

AUTHOR EMAIL ADDRESS: S. Oh (sroonter@hanyang.ac.kr); J. K. Jeong (jkjeong1@hanyang.ac.kr)

I. FABRICATION OF IGZO DEVICES

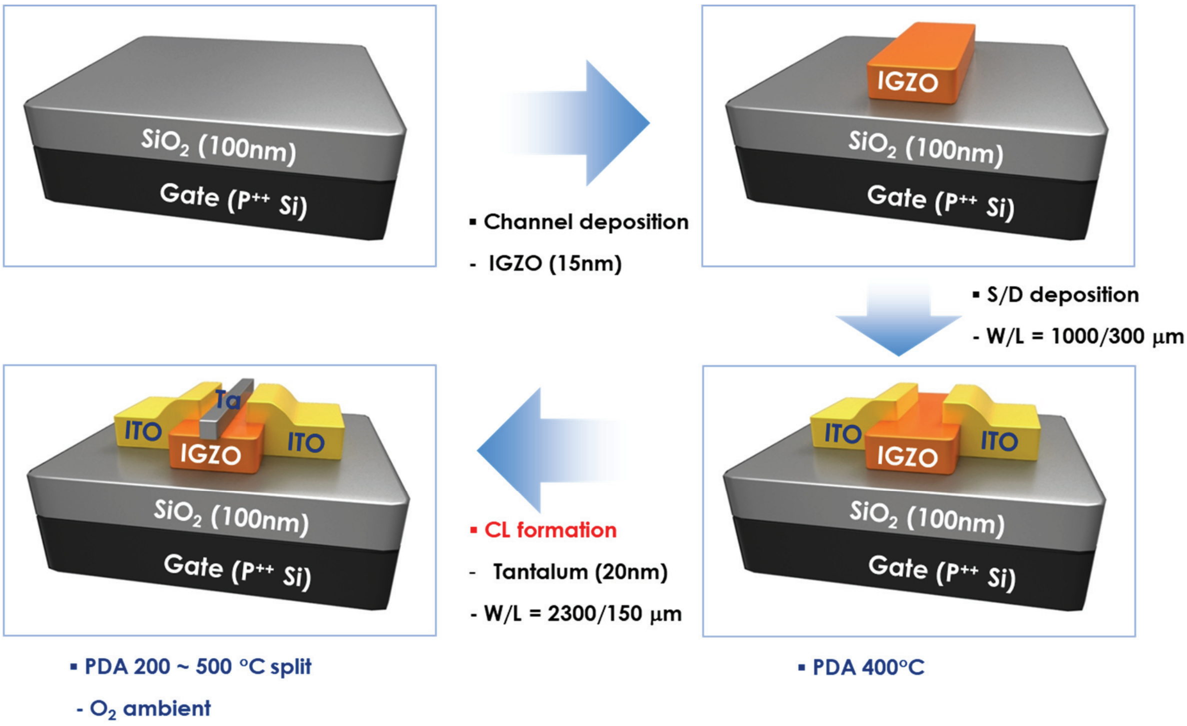

Figure S1. Procedure of Ta capped IGZO TFTs.

II. STRUCTURAL PROPERTY OF TANTALUM OXIDE FILMS

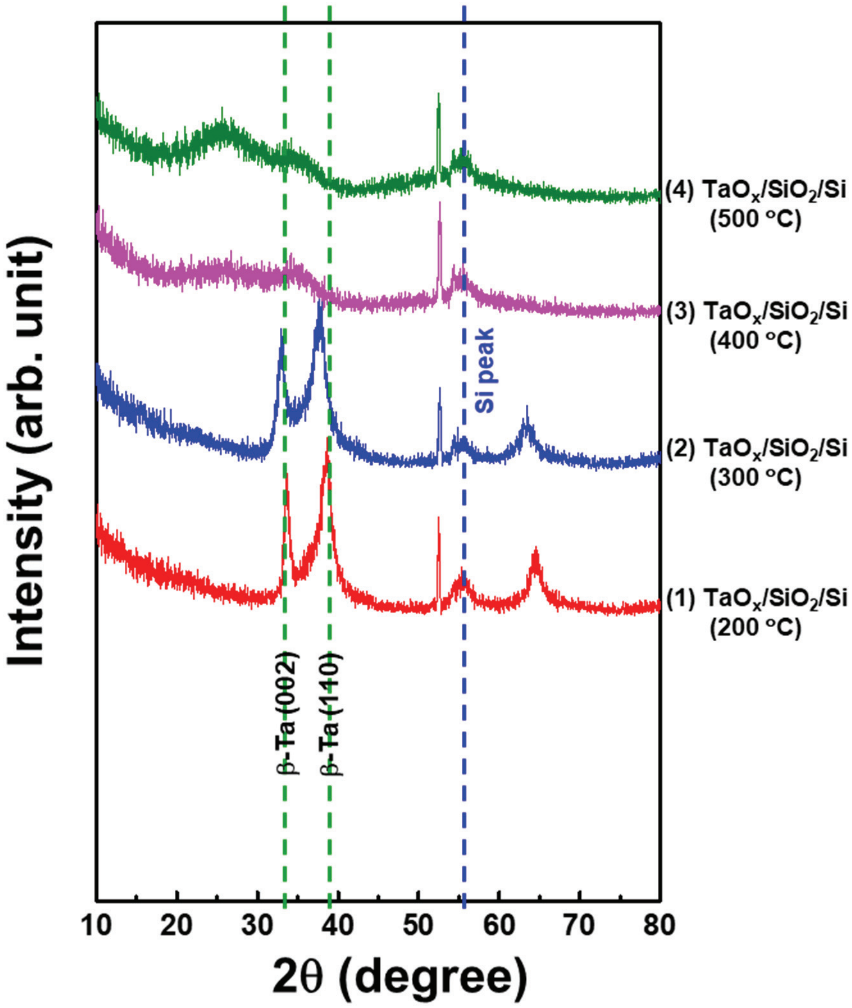

Figure S2. XRD spectra of the $TaO_x/SiO_2/Si$ stack without IGZO channel layer, after annealing at various temperatures under O_2 atmosphere.

III. CHEMICAL STATES OF IGZO FILMS

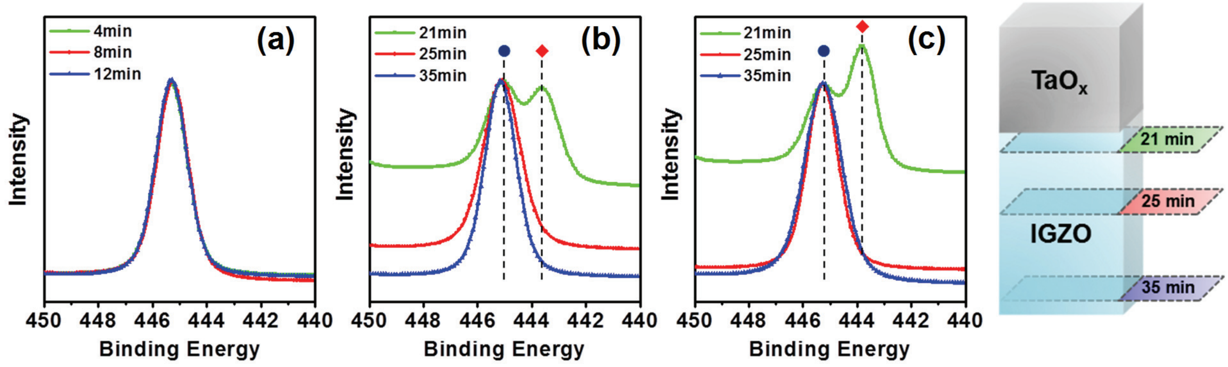

Figure S3. In *3d5/2* XPS spectra of the (a) reference IGZO sample, (b) Ta/IGZO sample annealed at 200 °C, and (c) Ta/IGZO sample annealed at 300 °C under O_2 atmosphere. Green line data for the Ta/IGZO samples annealed at 200 and 300 °C were taken from the IGZO film near TaO_x/IGZO interface. In *3d5/2* XP spectra can be de-convoluted into the In-O lattice and metallic In related sub peaks, which corresponds to 445.2 and 443.6 eV, respectively. Conversely, the bulk IGZO films far away the TaO_x/IGZO interface exhibit only the In-O lattice subpeak.

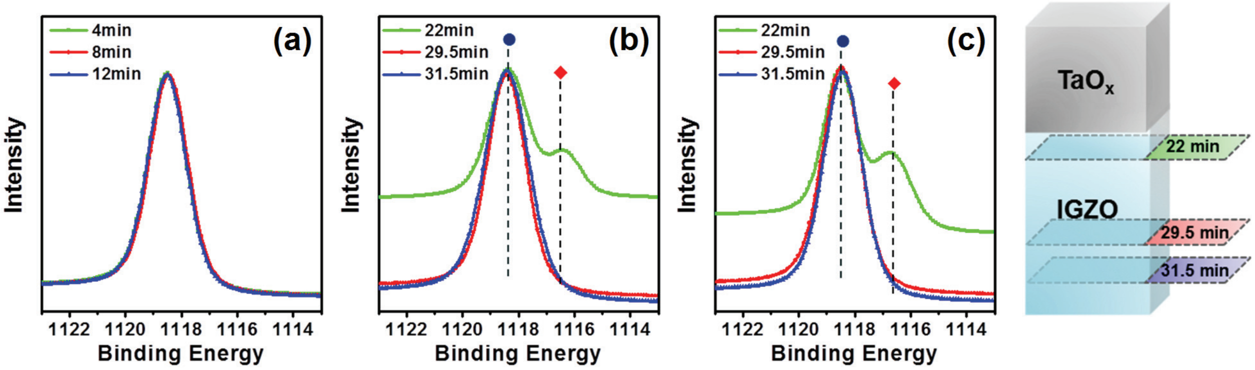

Figure S4. Ga *2p* XPS spectra of the (a) reference IGZO sample, (b) Ta/IGZO sample annealed at 200 °C, and (c) Ta/IGZO sample annealed at 300 °C under O_2 atmosphere. Green line data for the Ta/IGZO samples annealed at 200 and 300 °C were taken from the IGZO film near TaO_x/IGZO interface. Ga $2p_{3/2}$ XP spectra can be de-convoluted into the Ga-O lattice and metallic Ga related sub peaks, which corresponds to 1118.4 and 1116.4 eV, respectively. Conversely, the bulk IGZO films far away the TaO_x/IGZO interface exhibit only the Ga-O lattice subpeak.

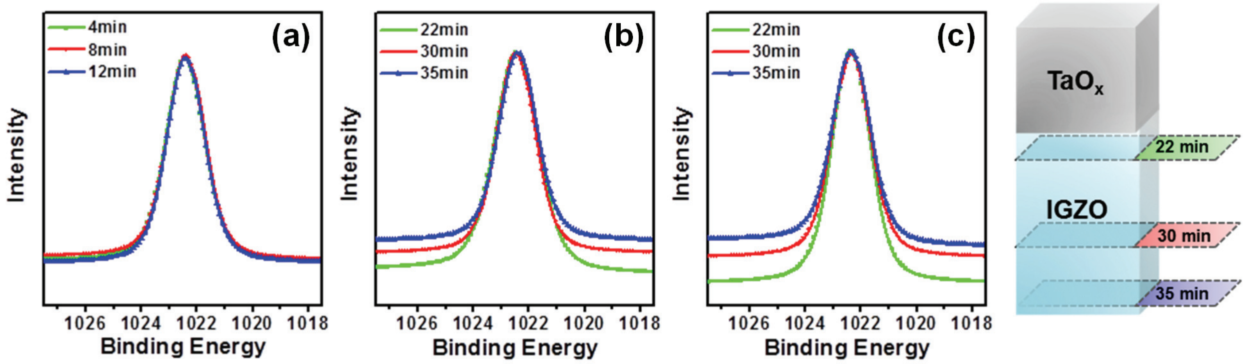

Figure S5. Zn *2p$_{3/2}$* XPS spectra of the (a) reference IGZO sample, (b) Ta/IGZO sample annealed at 200 °C, and (c) Ta/IGZO sample annealed at 300 °C under O_2 atmosphere. Green line data for the Ta/IGZO samples annealed at 200 and 300 °C were taken from the IGZO film near TaO_x/IGZO interface. Irrespectively of the depth position, Zn *2p$_{3/2}$* XP spectra had only Zn-O lattice peak at 1022.4 eV. It indicates that the chemical reaction of catalytic Ta and IGZO film causes the preferential breaking of In-O and Ga-O bonds.

IV. STRUCTURAL PROPERTY OF IGZO FILM

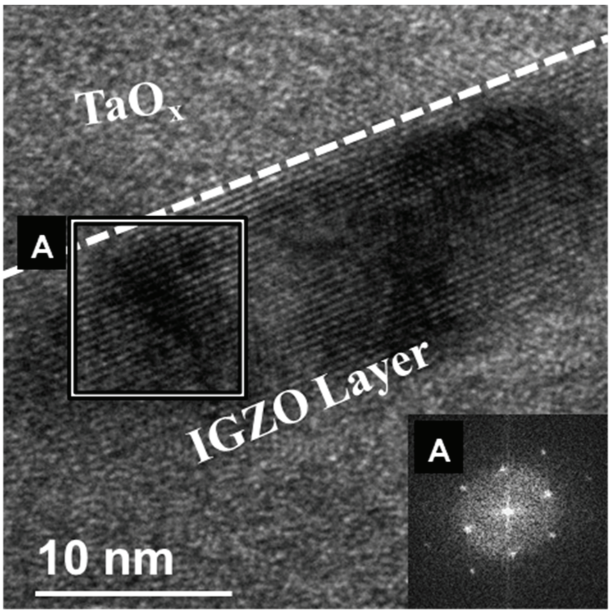

Figure S6. Cross-sectional TEM images of the IGZO layer with Ta catalytic layer after thermal annealing at 300 °C under O_2 atmosphere. (Partial crystallization is shown near the Ta/IGZO interface, when the IGZO thickness was 50nm.)

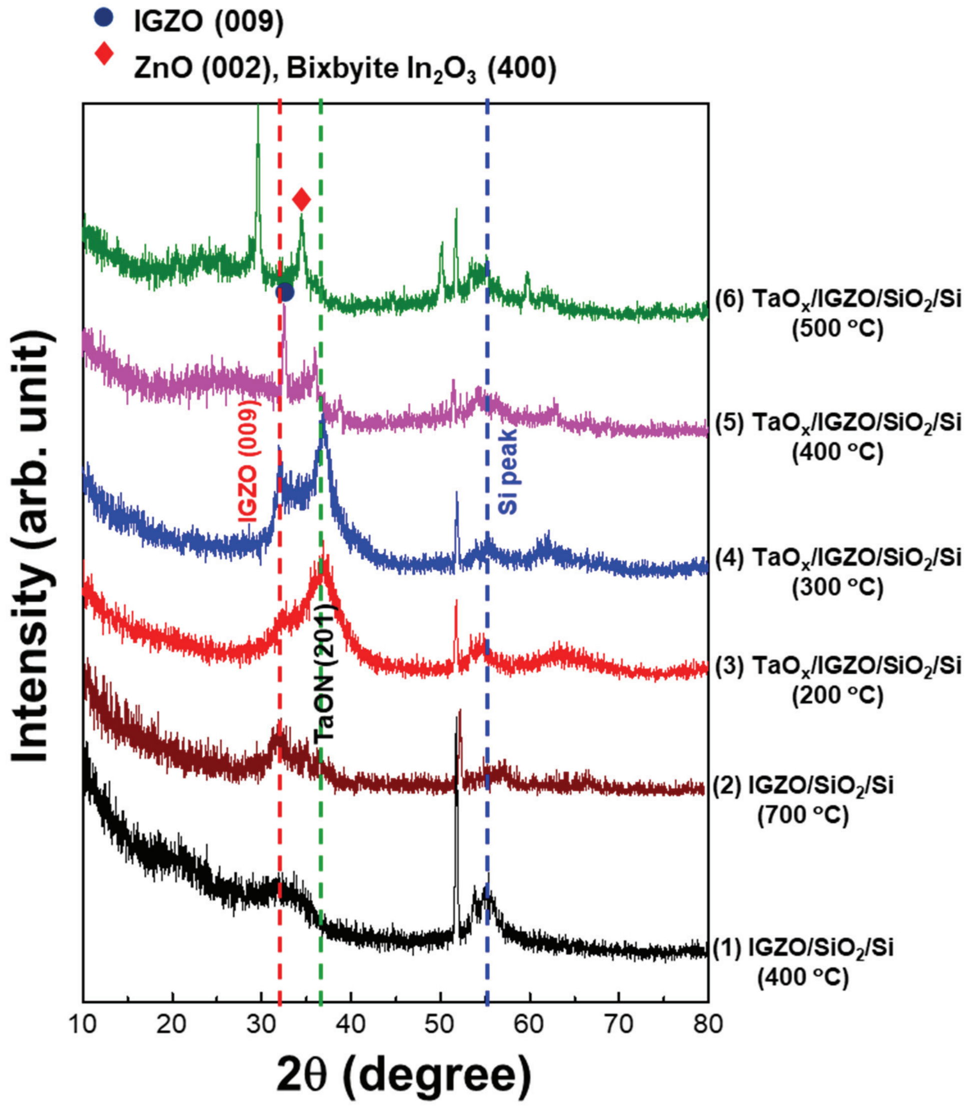

Figure S7. XRD spectra of the $TaO_x/SiO_2/Si$ stack, after annealing at various temperatures under N_2 atmosphere.

V. DEVICE PERFORMANCES OF IGZO TFTS

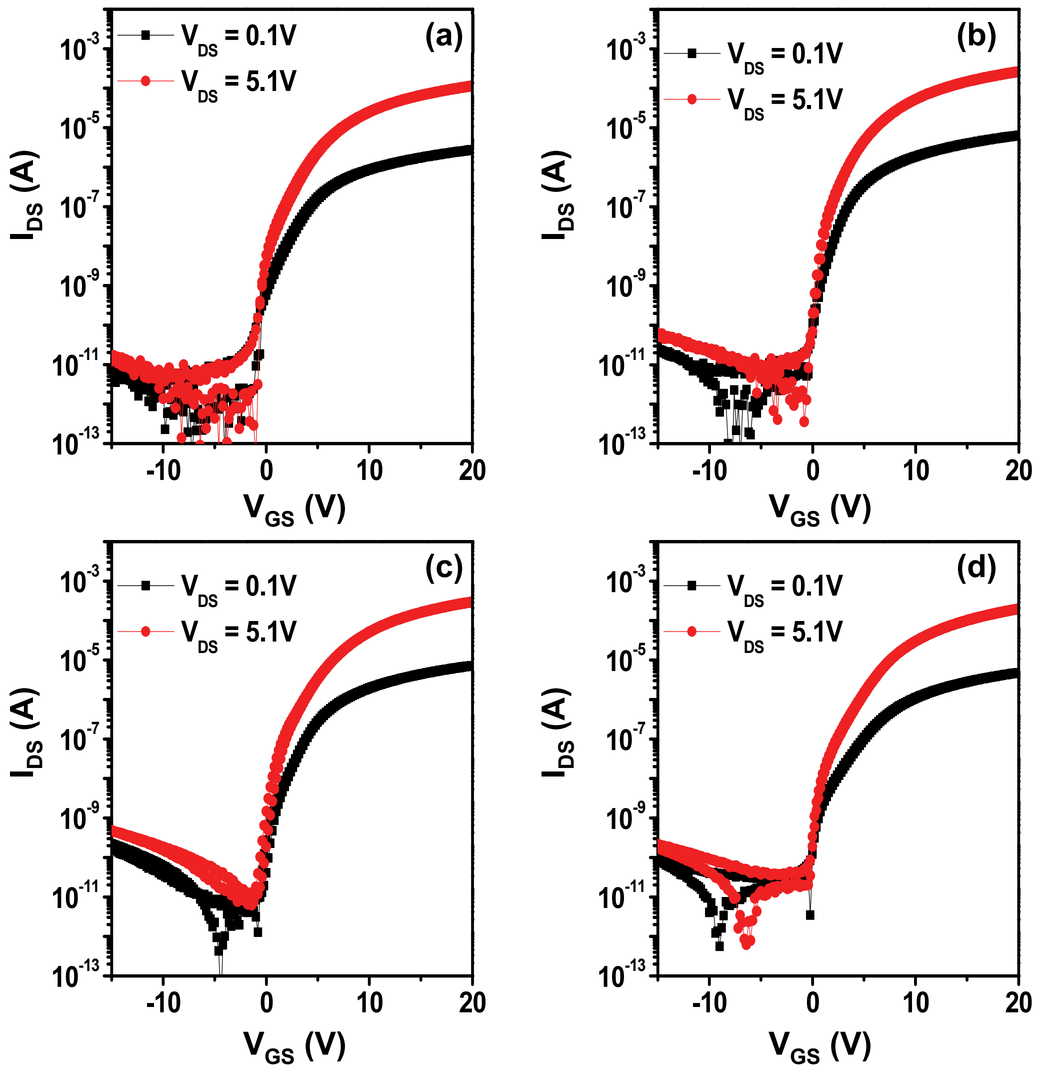

Figure S8. Transfer characteristics (I_D-V_{GS}) of the (a) reference IGZO device without the catalytic layer and the Ta-induced polycrystalline IGZO TFT annealed (b) 200 ˚C, (c) 300 ˚C, (d) 400 ˚C under N_2 atmosphere.

Table S1. Summary of the TFT device parameters with the reference IGZO and Ta/IGZO annealed at various temperatures under N_2 atmosphere. Average and standard deviation values are included.

**Samples**	**μ_{FE} (cm^2/Vs)**	**SS (V/decade)**	**V_{TH} (V)**	**$I_{ON/OFF}$**
(a) Control Device	18.1 ± 0.6	0.8 ± 0.1	0.9 ± 0.2	1.2×10^7
(b) Ta/ IGZO 200 °C	42.7 ± 3.4	0.5 ± 0.1	0.6 ± 0.4	3.1×10^7
(c) Ta/ IGZO 300 °C	51.7 ± 4.9	0.5 ± 0.1	1.0 ± 0.1	4.0×10^7
(d) Ta/ IGZO 400 °C	33.6 ± 2.9	0.6 ± 0.1	1.1 ± 0.5	1.9×10^7
